# Increasing Chinese EFL Learners’ Grit: The Role of Teacher Respect and Support

**DOI:** 10.3389/fpsyg.2022.880220

**Published:** 2022-05-03

**Authors:** Yumin Shen, Hongyu Guo

**Affiliations:** ^1^School of Foreign Languages, Zhejiang Gongshang University, Hangzhou, China; ^2^Graduate School of Education, University of Perpetual Help System DALTA, Las Piñas, Philippines

**Keywords:** teacher respect, EFL learners, China, grit, teacher support

## Abstract

Owing to the pivotal role of grit in scholastic success, factors that help learners become gritty are worth to be studied. Accordingly, this research sought to inspect the impact of teacher respect and teacher support on Chinese EFL learners’ grit. In doing so, three reliable measures of the variables were sent to 613 Chinese EFL learners. Using Spearman correlation tests, strong connections were discovered between teacher respect, teacher support, and Chinese EFL learners’ grit. Multiple regression analysis was then performed to inspect the role of teacher respect and teacher support in increasing Chinese EFL learners’ grit. As a result, both teacher respect and teacher support were found to be highly influential in increased learner grit. The limitations and implications are discussed.

## Introduction

Grit has been broadly viewed as a key predictor of learners’ scholastic success. Put simply, grit is believed to be of great help to learners who wish to attain desirable scholastic outcomes (Reed and Jeremiah, 2017; Liu and Wang, 2021). Because of this, learners’ level of grit seems to be of great importance to instructors in any instructional-learning context, including English as a foreign language (EFL) classes. Grit has been generally defined as “the amount of vigor that people spend in developing ambitious goals that will contribute to their development” (Duckworth et al., 2007, p. 1090). Likewise, learner grit has been conceptualized as the amount of passion, interest, and perseverance that learners demonstrate in attaining their scholastic objectives (Duckworth, 2016). Taking this definition into consideration, MacIntyre and Khajavy (2021) characterized L2 learner grit as individual learners’ determination to endure the hardships and difficulties of language learning in order to master the target language.

According to Wei et al. (2019), grittier L2 learners are more capable of mastering the second language as they pursue the learning process regardless of barriers, problems, and challenges. Lee (2020) further maintained that grit as a motivational variable also encourages L2 learners to eagerly communicate with their peers inside the classes. To him, L2 learners who constantly communicate in the second language are more likely to succeed in language courses. In a similar vein, Liu (2021) noted that grit drives L2 learners to persevere through the lengthy process of language learning. She also mentioned that this personal trait inspires L2 learners to enthusiastically engage in classroom activities, which helps them thrive in language learning environments. Due to the value of grit in the language learning process (Lee, 2020; Liu, 2021), inspecting the consequences and predictors of this construct seems crucial. To answer this necessity, some empirical investigations [e.g., Chang (2014),Hodge et al. (2018), and Yamashita (2018)] have been conducted into the academic consequences of language learners’ grit. Later, with the rise of positive psychology in language education, more research has been undertaken in this area [e.g., Khajavy et al. (2021), Li and Dewaele (2021), Resnik et al. (2021), Wang et al. (2021), and Zawodniak et al. (2021), among others]. Nevertheless, the predictors of language learners’ grit have remained elusive. Put simply, factors leading language learners to become gritty have been rarely explored [e.g., Gyamfi and Lai (2020),Yang (2021), and Yuan (2022)].

Both personal and interpersonal factors are believed to considerably enhance language learners’ grit (McCain, 2017; Muenks et al., 2017). An instance of interpersonal factor that may contribute to increased learner grit is teacher respect, which refers to the extent to which teachers acknowledge the significance or value of their learners (Hill, 1998). Webster (2003) also described teacher respect as “the degree to which teachers honor their learners and show esteem for their personality” (p. 2). As put forward by Hallinan (2008), teachers who treat learners with deference can inspire them to persevere in the learning process. Similarly, Celkan et al. (2015) postulated that learners who feel their teachers value their ideas and viewpoints are more inclined to continue their educational efforts. Another interpersonal variable that may lead learners to become gritty is the teacher support, which pertains to “the extent to which learners believe their teachers value and seek to establish personal relationships with them” (Chong et al., 2018, p. 2). According to Lei et al. (2018), supportive teachers are able to create an emotional learning climate that inspires learners to continue studying despite any hardships or difficulties. In a similar vein, Sadoughi and Hejazi (2021) stated that the provision of adequate support motivates learners to constantly take part in academic activities, which makes a great change in their learning outcomes.

Because of the pivotal role of teacher respect and teacher support in the language learning process (Celkan et al., 2015; Wang and Guan, 2020; Sadoughi and Hejazi, 2021), a sizable number of inquiries have been dedicated to these variables and their scholastic outcomes [e.g., Miller et al. (2017), Singer and Audley (2017), Feng et al. (2019), and Chang and Bangsri (2020), among others]. Yet, the impact of these variables on learner grit has been overlooked by previous studies. Simply put, to the authors’ knowledge, no empirical study, neither in language education nor in general education, has been conducted on the effects of teacher respect and teacher support on learner grit. This inquiry strives to fill the mentioned gaps by evaluating the role of teacher respect and teacher support in raising Chinese EFL learners’ grit.

## Literature Review

### Teacher Respect

The concept of respect, in a general sense, means to show consideration for others (Ellis, 1997). To Webster (2003), respect is “an all-encompassing state that determines how one individual views another and correspondingly how that person will interact with the other” (p. 3). With respect to this definition, Celkan et al. (2015) described teacher respect as the degree to which teachers show esteem for their learners’ personalities, individual differences, and viewpoints. Miller et al. (2017) also characterized this concept as the extent to which the thoughts and ideas of learners are valued by their teachers. As put forward by Patrick et al. (2007), a respectful learning atmosphere stimulates learners to engage in purposeful interactions with their peers and instructors. In this regard, Hallinan (2008) also declared that the acknowledgment of the worth of learners and their personal ideas encourages them to keep up learning.

To date, in spite of the value of teacher respect in instructional-learning contexts, only a small number of studies (Miller et al., 2017; Liang et al., 2020) have been done on this construct. For instance, Miller et al. (2017) examined the degree to which perceived teacher respect is correlated with teachers’ self-efficacy beliefs. To do this, 427 students and 51 teachers were requested to complete two reliable scales. Analyzing the gathered responses, the researchers found a direct connection between teachers’ self-efficacy beliefs and perceived teacher respect. Further, Liang et al. (2020) probed the association between a respectful classroom climate and learners’ feelings of depression. To do so, 467 learners were asked to answer two pre-developed scales. The evaluation of participants’ answers demonstrated a negative association between the respectful classroom climate and the learners’ level of depression.

### Teacher Support

Teacher support is generally concerned with the extent to which an individual teacher assists his or her learners in the learning process (Federici and Skaalvik, 2014). According to Pitzer and Skinner (2017), supportive teachers are those who care about their learners, satisfy their academic needs, and help them overcome the learning barriers. As put forward by Skinner et al. (2008), teacher support comprises three fundamental dimensions: *Support for autonomy*, *structure*, and *involvement*. The first dimension, support for autonomy, pertains to “teachers’ provision of choice, relevance, or respect to students” (Skinner et al., 2008, p. 767). The second dimension, structure, relates to the lucidity of expectations and eventualities (Jang et al., 2010). Involvement as the final dimension relates to how well teachers understand their learners and dedicate educational facilities to them (Lietaert et al., 2015).

Previous investigations into teacher support revealed that this communication behavior is tied to learners’ motivation (Vatankhah and Tanbakooei, 2014), academic effort (Dietrich et al., 2015), classroom engagement (Strati et al., 2017), and learning achievement (Hornstra et al., 2021). For one, Vatankhah and Tanbakooei (2014) studied the association between teacher support and learners’ extrinsic and intrinsic motivation. To this aim, two researcher-made scales were administered to 60 Iranian English language learners. Using correlational analyses, a strong relationship was found between teacher support and Iranian learners’ motivation. In another study, Dietrich et al. (2015) assessed the role of teacher support in learners’ amount of efforts. To do so, 1,155 German learners were asked to respond to two questionnaires. The results indicated that German learners perceived teacher support to be an influential factor in their level of academic effort.

### Learner Grit

The notion of “grit” generally refers to “effortful pursuit of goals despite setbacks” (Duckworth et al., 2007, p. 1088). Likewise, learner grit pertains to learners’ ongoing endeavors to reach their academic goals (Chang, 2014; Ivcevic and Brackett, 2014). In a comprehensive definition, Duckworth and Gross (2014) described learner grit as the amount of endurance and resistance learners demonstrate during the arduous process of learning. As Duckworth and Quinn (2009) mentioned, learner grit encompasses two underlying components, namely “*perseverance of effort*” and “*consistency of interests*”. Perseverance of effort pertains to the tenacious pursuit of objectives regardless of adversities and setbacks (Duckworth, 2016). Consistency of interest also refers the long-lasting passion of learners to meet their scholastic objectives (O’Neal et al., 2019). As noted by Bazelais et al. (2016), grit stimulates learners to take an active role in learning environments, which gradually contributes to their increased scholastic achievement. Similarly, Bennett et al. (2020) also asserted that gritty learners typically outperform their peers as they continually participate in learning tasks, no matter how challenging they are.

Owing to the value of grit in educational environments (Bazelais et al., 2016; Bennett et al., 2020), a large number of inquiries have been conducted on this personal trait [e.g., Teimouri et al. (2020); Wei et al. (2020), and Oxford and Khajavy (2021), to cite a few]. Likewise, the scholastic consequences of this variable have been widely explored [e.g., Chang (2014),Wang (2017), Yamashita (2018),Bliss and Jacobson (2020), and Li and Dewaele (2021), to cite a few]. Notwithstanding, the personal, interpersonal, and situational sources of learner grit have remained elusive. Moreover, compared to other potential sources, interpersonal factors have gained far less attention (Yuan, 2022). This inquiry sought to narrow the existing lacuna in the literature by inspecting the impact of two interpersonal factors, namely teacher respect and teacher support, on Chinese EFL learners’ grit. To do so, two research questions were formulated, as below:

a. Are there any significant correlations between teacher respect, teacher support, and Chinese EFL learners’ grit?
b. To what extent can teacher respect and teacher support significantly predict Chinese EFL learners’ grit?

## Materials and Methods

### Participants

Maximum variation sampling, which is also called maximum diversity sampling, was utilized to choose the respondents of this investigation. Maximum variation sampling is an instance of a “*purposive sampling strategy*” in which “researchers attempt to collect data from the widest range of perspectives possible about a certain topic” (Emmel, 2013, p. 34). Relying on the principles of this strategy, 613 Chinese EFL learners were selected from different cities and provinces of this country (i.e., Anhui, Beijing, Fujian, Guangdong, Hebei, Heilongjiang, Henan, Jilin, Shandong, Shanghai, Shanxi, and Zhejiang). The sample consisted of 553 females and 60 males with different age levels (*Range* = 15–45, *Mean* = 20.87, *SD* = 3.65). Half of the participants were Ph.D. candidates (*N* = 121) or MA students (*N* = 186), and the rest were undergraduate students (*N* = 306). It is worth noting that taking part in this investigation was entirely voluntary.

### Instruments

#### L2 Grit Scale

To examine the extent to which Chinese EFL learners perceive themselves to be gritty, “*L2 Grit Scale*” (Teimouri et al., 2020) was employed. The scale consists of nine items to which respondents score on a 5-point Likert scale (1 = Not like me at all, 5 = Very much like me). The following are three instances of L2 grit scale: item (1) “*I am a diligent English language learner*”, item (3) “*When it comes to English, I am a hard-working learner*”, and item (5) “*Now that I have decided to learn English, nothing can prevent me from reaching this goal*”. In this inquiry, the reliability of L2 grit scale was measured to be 0.72.

#### Teacher Respect Scale

To evaluate perceived teacher respect, “Teacher Respect Scale (TRS),” developed by Celkan et al. (2015), was utilized. The TRS encompasses 15 closed-ended items, each assessed on a 5-point Likert-type scale. Some of these items are: item (4) “*The instructor is fair and reasonable*,” item (7) “*The instructor is sincere*,” and item (12) “*The instructor inspires students to work.*” The reliability of TRS was 0.97 in this research.

#### Teacher Support Scale

The “Teacher Support Scale (TSS)” (Metheny et al., 2008), which is a reliable measure of perceived teacher support, was utilized to assess the extent to which Chinese EFL learners are provided with teacher support. The TSS includes 21 items, some of which are mentioned hereafter: item (9) “*My English teacher pushes me to succeed*”, item (16) “*My English teacher encourages me to learn*” and item (18) “*My English teacher supports my goals for the future.*” The reliability index of TSS for this inquiry was 0.98.

### Procedure

Initially, through WeChat messenger, the electronic version of the consent form was sent to 850 Chinese EFL learners. Of those, 613 learners agreed to take part in this research. Then, the above-mentioned scales, including the L2 grit scale, TRS, and TSS, were distributed among respondents using the Wenjuanxing platform. To guarantee the accuracy and credibility of answers, all respondents were briefed on how to rate the items in the questionnaires. The answers were fully received in 20 days. The respondents’ answers were double-checked to detect and exclude any probable mistakes. Afterward, the correlations between teacher respect, teacher support, and learner grit were evaluated *via* the Spearman Rho correlation. Following that, multiple regression analysis was performed to identify how much of the variance in the Chinese EFL learners’ grit can be attributed to teacher respect and teacher support.

## Results

To begin, Kolmogorov-Smirnov tests were run to assess the normality of the collected data. The results of the tests are presented in the table below (Table 1).

**TABLE 1:** Results of Kolmogorov-Smirnov tests.

	Kolmogorov-Smirnov		
	Statistic	df	Sig.
L2 Grit Scale	0.096	613	0.000
Teacher respect scale (TRS)	0.176	613	0.000
Teacher support scale (TSS)	0.127	613	0.000

As demonstrated in Table 1, the distribution of data was found to be abnormal for all of the questionnaires (i.e., L2 grit scale, TRS, TSS). Because of this, non-parametric tests were used to analyze the gathered responses. Initially, the Spearman correlation test was utilized to evaluate the associations between teacher respect, teacher support, and learner grit. The outcomes of the Spearman correlation test are shown in Table 2.

**TABLE 2:** The outcomes of the Spearman correlation test.

	Teacher respect	Teacher support	Learner grit	
Teacher respect	Correlation coefficient	1.000	0.739**	0.245**
	Sig. (2-tailed)		0.000	0.000
	N	613	613	613
Teacher support	Correlation coefficient	0.739**	1.000	0.300**
	Sig. (2-tailed)	0.000	.	0.000
	N	613	613	613
Learner grit	Correlation coefficient	0.245**	0.300**	1.000
	Sig. (2-tailed)	0.000	0.000	.
	N	613	613	613

**Correlation is significant at the 0.01 level (2-tailed).

As shown in Table 2, a positive and strong correlation was discovered between teacher respect and teacher support (*r* = 0.739, *n* = 613, *p* = 0.000, α = 0.01). The results of Spearman correlation test also indicated a favorable relationship between teacher respect and learner grit (*r* = 0.245, *n* = 613, *p* = 0.000, α = 0.01). Likewise, a significant association was found between teacher support and learner grit (*r* = 0.300, *n* = 613, *p* = 0.000, α = 0.01). Taken together, the results of Spearman correlation test revealed that any enhancement in the indices of teacher respect and teacher support will cause an enhancement in the index of learner grit.

Then, multiple regression analysis was run to inspect the power of teacher respect and teacher support in predicting Chinese EFL learners’ grit. The results of multiple regression analysis are demonstrated hereunder (Table 3).

**TABLE 3:** Model summary for teacher respect, teacher support, and learner grit.

Model	R	R square	Adjusted R square	Std. error of the estimate	Durbin-Watson
1	0.279^a^	0.88^b^	0.85	5.71	1

*^a^Predictors: Teacher Respect, Teacher Support.*

*^b^ Dependent Variable: Learner Grit.*

As Table 3 revealed, 88% of the variance in Chinese learners’ grit can be attributed to teacher respect and teacher support. The Beta coefficient table was then studied to find out which of the independent variables, including teacher respect and teacher support, contributed more to increased learner grit.

According to the beta column (Table 4), teacher support, with a beta coefficient of 0.24, made the largest contribution to increased learner grit. In fact, teacher support was found to be the stronger predictor of Chinese EFL learners’ grit.

**TABLE 4:** The coefficients for teacher respect, teacher support, and learner grit.

Model		Unstandardized coefficients		Standardized coefficients	*t*	Sig.
	*B*	Std. error	Beta			
1	(Independent)	20.805	1.200		17.341	0.000
	Teacher respect	0.028	0.030	0.160	1.951	0.000
	Teacher support	0.081	0.021	0.247	3.876	0.000

## Discussion

The current research aimed to uncover the degree to which Chinese EFL learners’ grit is correlated with teacher respect and teacher support. The Spearman correlation analysis documented, first, a positive correlation between teacher respect and learner grit, and second, a favorable connection between teacher support and learner grit. In addition, a strong link was discovered between teacher respect and teacher support. Concerning the positive association between teacher respect and Chinese EFL learners’ grit, it is encouraging to compare this outcome with that discovered by Miller et al. (2017), who reported a favorable connection between perceived teacher respect and students’ level of perseverance and effort. This outcome also indirectly supports Gyamfi and Lai’s (2020) findings, which indicated that teachers’ positive behaviors are strongly tied to student grit. Regarding the favorable connection between teacher support and Chinese learners’ grit, it is fair to compare this result with that of Dietrich et al. (2015), who discovered that teacher support is highly associated with students’ constant efforts. This result also confirms the findings of Feng et al. (2019), who found that both parental and teacher support are significantly correlated with the amount of students’ persistence and effort.

This research also sought to identify the role of teacher respect and teacher support in increasing Chinese learners’ grit. To put it another way, the current study sought to determine whether improvements in the amount of teacher respect and teacher support contribute to increased learner grit. As the outcomes of regression analyses revealed, both teacher respect and teacher support were found to be influential in increasing Chinese EFL learners’ grit. The positive influence of teacher respect on Chinese EFL learners’ grit may be justified by the fact that students who are respected and valued by their instructors tend to pursue the learning process despite the hardships and challenges (Hallinan, 2008; Celkan et al., 2015). Besides, the favorable effect of teacher support on Chinese EFL learners’ grit can be supported by the ideas of Lei et al. (2018), who stated that a supportive learning climate inspires students to keep trying regardless of barriers. This result may also have something to do with the fact that the provision of support to learners motivates them to persevere through the learning process (Sadoughi and Hejazi, 2021).

## Conclusion

The current research was an endeavor to determine the role of teacher respect and teacher support in increasing Chinese EFL learners’ grit. The outcomes of Spearman Rho correlation and multiple regression analyses evinced that teacher respect and teacher support can remarkably increase Chinese EFL learners’ grit. Simply put, these interpersonal behaviors help Chinese EFL learners become gritty. This outcome appears to be insightful for all English language teachers, notably those who are currently teaching in an EFL context. Building upon the results of this study, EFL teachers need to support their learners and show esteem for their viewpoints in order to make them grittier. Likewise, this result seems to be enlightening for teacher trainers as some of the EFL teachers do not know how to effectively support their learners in the laborious process of learning. Thus, teacher trainers are expected to brief their trainees on how to support pupils in classroom contexts. Given the pivotal role of teacher respect in increasing learner grit, they are also required to make their trainees aware of the value of this communication behavior.

Finally, a number of limitations need to be mentioned concerning the current research. First, a quantitative method was adopted to perform this inquiry. Accordingly, only close-ended scales were employed to delve into participants’ viewpoints. Future investigations into this subject are advised to adopt a different method of research (i.e., qualitative research, mixed-method research) and different means of data collection (e.g., narrative inquiry, open-ended scale, etc.) to come up with more comprehensive outcomes. Second, this research was fully conducted in China which is an EFL country. Thus, the outcomes may not be generalizable to English as a second language (ESL) countries. As such, it is recommended that future research on learner grit be implemented in an ESL country. Third, in this research, the probable effects of gender, age, and proficiency level on the correlation of the variables were overlooked. A future study assessing the effects of these factors would be interesting.

## Data Availability Statement

The original contributions presented in the study are included in the article/Supplementary Material, further inquiries can be directed to the corresponding author.

## Ethics Statement

The studies involving human participants were reviewed and approved by Zhejiang Gongshang University Academic Ethics Committee. The patients/participants provided their written informed consent to participate in this study.

## Author Contributions

Both authors listed have made a substantial, direct, and intellectual contribution to the work, and approved it for publication.

## Conflict of Interest

The authors declare that the research was conducted in the absence of any commercial or financial relationships that could be construed as a potential conflict of interest.

## Publisher’s Note

All claims expressed in this article are solely those of the authors and do not necessarily represent those of their affiliated organizations, or those of the publisher, the editors and the reviewers. Any product that may be evaluated in this article, or claim that may be made by its manufacturer, is not guaranteed or endorsed by the publisher.
