# Supplementary material for: Increasing Chinese EFL Learners’ Grit: The Role of Teacher Respect and Support
Source: Front Psychol. 2022 May 3;13:880220. doi: 10.3389/fpsyg.2022.880220 (PMC9113390; doi:10.3389/fpsyg.2022.880220)
Supplement: Supplementary file 1 [file Data_Sheet_1.docx]

**Appendix**

**1. L2 Grit**

Instruction: Please read the following list carefully. The items of this scale measure your L2 Grit. All items are scored on a 5-point frequency rating scale ranging from 1 (Not like me at all) to 5 (Very much like me).

1. I am a diligent English language learner.

2. My interests in learning English change from year to year.

3. When it comes to English, I am a hard-working learner.

4. I think I have lost my interest in learning English.

5. Now that I have decided to learn English, nothing can prevent me from reaching this goal.

6. I will not allow anything to stop me from my progress in learning English.

7. I am not as interested in learning English as I used to be.

8. I was obsessed with learning English in the past but have lost interest recently.

9. I put much time and effort into improving my English language weaknesses.

**2. Teacher Respect**

Instruction: Please read the following list carefully. For each statement, select the response that best represents your Agreement or Disagreement. Ranging from 1 (Strongly disagree) to 5 (Strongly agree).

1. The instructor tries to reach a class agreement rather than impose top down rules.

2. The instructor favors interaction in class and hence allows students to voice their ideas.

3. The instructor acknowledges the physical needs of students.

4. The instructor is fair and reasonable.

5. The instructor has a sense of humor.

6. The instructor provides a secure learning space.

7. The instructor is sincere.

8. The instructor treats students caringly.

9. The instructor provides opportunities for choice.

10. The instructor treats each student as an individual.

11. The instructor demonstrates professionalism.

12. The instructor inspires students to work.

13. The instructor displays a positive attitude toward students.

14. The instructor is flexible.

15. The instructor is fair in treating each and every student.

**3. Teacher Support**

Instruction: Please read the following list carefully. For each statement, select the response that best represents your Agreement or Disagreement. Ranging from 1 (Strongly disagree) to 5 (Strongly agree).

My English teacher...

1. expects me to work hard in school.

2. tries to answer my questions.

3. is interested in my future.

4. takes the time to help me get better grades.

5. thinks I am a hard worker.

6. is helpful when I have questions about career issues.

7. is helpful when I have questions about school issues.

8. would tell other people good things about me.

9. pushes me to succeed.

10. challenges me to think about my future goals.

11. believes I am smart.

12. helps me understand my strengths.

13. wants me to do well in school.

14. enjoys having me in their classes.

15. cares about what happens to me.

16. encourages me to learn.

17. thinks I should continue my education after high school.

18. supports my goals for the future.

19. will listen if I want to talk about a problem.

20. is easy to talk to about school things.

21. is easy to talk to about things besides school.
